# Comparing the EQ-5D-5L and stroke impact scale 2.0 in stroke patients: an analysis of measurement properties

**DOI:** 10.1186/s12955-024-02252-z

**Published:** 2024-06-05

**Authors:** Juliana Schmidt, Juliane Andrea Düvel, Svenja Elkenkamp, Wolfgang Greiner

**Affiliations:** Department of Health Economics and Health Care Management, School of Public Health, Bielefeld University, Bielefeld, Germany

**Keywords:** EQ-5D-5L, SIS, Stroke, Health-related quality of life, Psychometrics

## Abstract

**Background:**

Stroke has evolved to become a chronic disease and a major public health challenge. To adequately capture the full disease burden of stroke patients, the assessment of health-related quality of life (HRQoL) and thus the performance of respective measures is increasingly relevant. The aim of this analysis was to compare the measurement properties of two self-report instruments, the EQ-5D-5L and the Stroke Impact Scale 2.0.

**Methods:**

The data used for the analysis was derived from a quasi-experimental case management study for mildly to moderately affected incident stroke and transient ischemic attack (TIA) patients aged ≥ 18 in Germany. Data was collected patient-individually at 3, 6 and 12 months after initial stroke. The EQ-5D-5L and SIS 2.0 were compared in terms of feasibility, ceiling and floor effects, responsiveness and known-groups validity (Kruskal-Wallis H and Wilcoxon rank-sum test).

**Results:**

A response for all three follow-ups is available for *n* = 855 patients. The feasibility of the EQ-5D-5L is determined as good (completion rate: 96.4–96.6%, ≥ one item missing: 3.2 − 3.3%), whereas the SIS 2.0 is moderately feasible (overall completion rate: 44.9–46.1%, ≥ one item missing in domains: 4.7 − 28.7%). The SIS 2.0 shows substantial ceiling effects in comparable domains (physical function: 10.4 − 13%, others: 3.5–31.3%) which are mainly larger than ceiling effects in the EQ-5D-5L index (17.1–21.5%). In terms of responsiveness, the EQ-5D-5L shows small to moderate change while the SIS 2.0 presents with moderate to large responsiveness. The EQ-5D-5L index, mobility, usual activities and Visual Analogue Scale show known-groups validity (*p* < 0.05). Content-related domains of the SIS 2.0 show known-groups validity as well (*p* < 0.05). However, it is compromised in the emotion domain in both measures (*p* > 0.05).

**Conclusions:**

The EQ-5D-5L seems to be slightly more suitable for this cohort. Nonetheless, the results of both measures indicate limited suitability for TIA patients. Large-scale studies concerning responsiveness and known-groups validity are encouraged.

**Trial registration:**

The study was registered in the German Clinical Trials Register, retrospective registration on 21.09.2022. Registration ID: DRKS00030297.

**Supplementary Information:**

The online version contains supplementary material available at 10.1186/s12955-024-02252-z.

## Background

For the last three decades, stroke has been top-ranked in the list of the largest causes of Disability-Adjusted Life Years and leading causes of death worldwide [1, 2]. High rates of recurrence additionally contribute to a high burden of disease [3]. Although the incidence of stroke is declining, improved survivorship and an ageing population are leading to a higher prevalence of stroke, sequelae and an increasing need for care. Hence, stroke is considered a chronic disease, of which the burden is expected to increase significantly in the future [2, 4–6].

However, the disability inherently accompanied by stroke as estimated by clinical measures often do not portray the full range of medical, familial, social and professional impairments and limitations faced post stroke. Consequently, patient-reported outcomes such as measures of Health-Related Quality of Life (HRQoL) have increasingly been integrated into clinical studies and post-stroke assessment [7–13]. Exemplary of such are the EQ-5D-5L (5L) and the Stroke Impact Scale 2.0 (SIS). The former was developed by the EuroQol Group, with the aim of establishing a standardized, generic instrument for describing and valuing HRQoL in a wide variety of diseases and health care sectors [14, 15]. The 5L is a frequently used measure [16], for which adequate psychometric performance has been demonstrated, both in the general population and for specific diseases, such as stroke [17–22]. However, it has been criticized that the 5L does not assess the breadth of impairments, which occur post stroke and that are consequently relevant for a comprehensive assessment of HRQoL in stroke survivors [23]. A measure which addresses a wider range of possible impairments after stroke is the SIS. It is a disease-specific instrument developed for patients with mild to moderate stroke. Alike the 5L, evidence suggests that the SIS is a valid instrument for the assessment of HRQoL [24, 25].

Although various measures have been utilized, discussions and studies have not come to a consensus on a standard measure of HRQoL in stroke. Both generic and stroke-specific instruments bare potentials and limitations in terms of psychometric properties [8, 26, 27]. This highlights the need for analyses comparing different measures of HRQoL within the same population [28] to enable evidence-based methodological decisions for future research. The investment in psychometric studies remains important in stroke research [7]. To the best of our knowledge, there has yet been no comparison of the generic 5L and the disease-specific SIS to determine the adequacy for the assessment of HRQoL in stroke patients. Thus, this analysis aimed at comparing the measurement properties of the 5L and the SIS in stroke patients undergoing case-management in Germany, by evaluating feasibility, ceiling and floor effects, responsiveness and known-groups validity.

## Methods

### Study sample

The data used for the current analysis was derived from a quasi-experimental case management study for stroke and transient ischemic attack (TIA) patients in Germany and collected between 06/2018 and 03/2021. Owing to the study design (matching of controls) only patients assigned to the intervention group were included in this analysis. Patients’ eligibility criteria include: (i) ICD-10 codes I60 (Subarachnoid haemorrhage), I61 (Intracerebral haemorrhage), I62 (Other non-traumatic intracerebral haemorrhage), I63 (Ischemic stroke), I64 (Stroke, not described as haemorrhage or infarction) and G45 (Transient ischemic attack), (ii) aged ≥ 18 years, (iii) modified Rankin Scale (mRS) 0–4 at baseline (stroke unit) (out of 6; the higher the score the higher the disability), (iv) long term care grade < 4 (out of 5; the higher the grade the greater the need for assistance), (v) first ever stroke. The following criteria resulted in exclusion of patients: (i) in-patient long-term care (ii) severe comorbidities (e.g. malignant neoplasm, Alzheimer’s disease or other neurogenerative diseases, organic mental disorders). Methods of the study have been reported in detail elsewhere [29]. Patients with a confirmed stroke or TIA diagnosis according to claims data were included in the analysis. Potential biases of the COVID-19 pandemic on the study participants’ HRQoL such as reduced HRQoL outcomes due to restrictions in daily life and poorer psychological health outcomes [30, 31], were analyzed separately and rejected by statistical comparison of the two cohorts (pre-pandemic vs. pandemic).

### Outcome measures

Patients’ post-stroke level of disability was assessed by the clinician-reported mRS [32, 33] which is a validated stroke- and neurology-specific scale ranging from 0 (no symptoms at all) to 6 (death) [34]. Their performance in activities of daily living (ADL) was measured by the Barthel Index (BI). The BI is a validated measure scoring 0 (unable to carry out ADL) − 100 (able to carry out ADL) [35, 36]. For analysis purposes, the BI scoring was categorized into three groups: i) ≤ 50, ii) 51–75, iii) ≥ 76 [37]. Self-reported HRQoL based on the 5L and the SIS was collected during rehabilitation at 3 (t_1_), within the home setting at 6 (t_2_) [38] and at the end of intervention phase at 12 (t_3_) months after initial stroke. A study nurse reminded patients to respond within three weeks after sending out the paper-based questionnaires. The descriptive system of the 5L covers five dimensions: mobility (MO), self-care (SC), usual activities (UA), pain/discomfort (PD) and anxiety/depression (AD). Each dimension can be described by five levels of problems: 1 - no problems, 2 - mild problems, 3 – moderate problems, 4 – severe problems and 5 - extreme problems. A total of 3125 possible health states can be distinguished by the 5L, which can be described in a five-digit profile [39]. Next to the descriptive system a Visual Analogue Scale (EQ VAS) is included in the 5L. For the EQ VAS, respondents were asked to assess their overall health on a scale from 0 (worst imaginable health) to 100 (best imaginable health) [40]. The German preference-based value set by Ludwig and colleagues was used to calculate the 5L index. The 5L index values for the German value set range from − 0.661 to 1 [41]. The SIS consists of 64 items comprised into eight domains: (i) strength, (ii) hand function, (iii) mobility, (iv) (instrumental) activities of daily living (ADL/IADL), (v) memory, (vi) communication, (vii) emotion, (viii) participation and role function. Each item is rated by a five-point Likert scale indicating the extend of disability and difficulty faced by the patient: (i) could not do it at all, (ii) very difficult, (iii) somewhat difficult, (iv) a little difficult, (v) not difficult at all. For each domain an aggregate score, based on a scoring algorithm, can be calculated [42]. Following this method, each domain generates scores in the range of 0 to 100, representing the worst and the best score possible, respectively [24]. Moreover, a physical function domain can be calculated by averaging across the domains: strength, hand function, ADL/IADL and mobility [43]]. Apart from the named domains, the SIS includes the assessment of patients’ global perception of recovery in the form of a VAS that is different from the EQ VAS. On the SIS VAS, 0 means not recovered and 100 is considered as full recovery [24].

### Data analysis

Initially, descriptive analysis of socio-demographic characteristics was carried out. The analysis of characteristics was based on the sample of patients, which have returned the 5L from t_1_ to t_3_. Psychometric analysis was based on follow-up data from t_1_ to t_3_. By generating a physical function domain for the SIS, comparison to the 5L index was facilitated, as the focus of the 5L dimensions lies primarily within physical aspects (MO, SC, UA, PD).

### Feasibility

Feasibility was determined by absolute numbers and percentage of missing values per dimension and completion rate of the whole 5L questionnaire. The corresponding EQ VAS was analyzed in terms of completion rate. Likewise, the domains and individual items of the SIS, were assessed by absolute numbers and percentage of missing scores. Additionally, the domains and the overall questionnaire were assessed by completion rate. In order to compare the feasibility of both measures, the 5L index was contrasted to each domain of the SIS. Furthermore, feasibility of the measures respective VAS was compared. Patients who have returned the instrument of interest at all time points were considered for the analysis of feasibility.

### Floor and ceiling effects

For the 5L, profiles of “11111” and “55555” were considered for floor and ceiling effects, respectively. Analogously, the calculated score of 0 or 100 indicated either floor or ceiling effects in the domains of the SIS, the SIS VAS and the EQ VAS. According to Terwee et al. (2007), a threshold of > 15% of the respondents achieving the highest or lowest possible score, served as indication for a ceiling or floor effect [44]. As the analysis of floor and ceiling effects requires completed instruments or domains (complete case analysis), patients with a complete 5L index or SIS domains at the respective time points were considered for the analyses.

### Responsiveness

For the responsiveness analysis the BI was utilized as an external anchor. Since no “gold standard” has been established, relevant change was determined by minimal clinically important difference (MCID) as estimated by Hsieh and colleagues [45] and adjusted by the team of Golicki [18]. A change of the BI by ≥ 9.25 points between two time points was considered as either improvement or as deterioration. Patients with a change in the BI < 9.25 points between two time points were regarded as stable. In the analysis, responsiveness was determined by Effect Size (ES) and Standardized Response Mean (SRM). Both ES (mean change in scores, divided by the standard deviation of baseline scores [46]) and SRM (mean change in scores divided by the standard deviation of the change score [47]) were interpreted according to commonly accepted criteria: > 0.8 large, 0.5–0.8 moderate and < 0.5 small [48]. For the assessment of responsiveness of the 5L index, the SIS physical function domain and respective VAS, a complete case analysis from t_1_, to t_3_ was used to ensure the analysis of one cohort.

### Known-groups validity

Due to non-normality of the data, nonparametric tests were used to determine known-groups validity: Kruskal-Wallis H test and Wilcoxon rank-sum test (two-sided due to non-uniform directions of scores between groups according to instrument). If the omnibus test produced statistically significant results, a nonparametric post-hoc Dunn-Bonferroni test was performed. Complete cases were used for this analysis. It was hypothesized that HRQoL measured by the 5L, SIS, their respective VAS and select dimensions would differ by age [49–51], sex [50, 52], mRS [49, 51, 53], BI [54–56] and type of stroke [57]. For the analyses, age groups were categorized as follows: ≤ 44, 45–49, 50–54, 55–59, 60–64, 65–69, 70–74, 75–79, 80–84 and ≥ 85. BI was grouped into five categories: 0–24, 25–49, 50–74, 75–99 and 100. Known-groups validity was examined cross-sectionally by including patients with complete questionnaires or domains at t_1_. To contrast the 5L and the SIS, the following comparisons were made: 5L index vs. SIS physical function domain; EQ VAS vs. SIS VAS; 5L AD vs. SIS depression; 5L UA vs. SIS ADL/IADL; 5L MO vs. SIS mobility.

To avoid selection bias, for each above-mentioned analysis, we compared patient characteristics of patients with complete and incomplete questionnaires before carrying out complete case analyses. Statistical analyses and reporting were guided by the COSMIN design and reporting standards [58]. All statistical analyses were performed with R Statistical Software version 4.3.1 [59] using the following packages: DescTool [60], tidyverse [61] and FSA [62]. Results were considered statistically significant at *p* < 0.05 and all statistical tests were two-sided.

## Results

The response rate for both measures at the respective time points is 73% (t_1_), 72% (t_2_) and 69% (t_3_). Complete follow-up data, in the sense that all three questionnaires of the SIS were returned, is available for *n* = 856 patients. Data of patients that have completed all three follow-ups of the 5L is available for *n* = 855 patients. An overview of the demographic and disease characteristics based on the cohort, which returned all 5L assessments is provided in Table 1.

**Table 1:** Demographic characteristics of the study sample at baseline

Characteristics (*n* = 855)	
Age, *years*	
Mean (SD)	69.91 (12.17)
Range	27–93
Sex, *n (%)*	
Female	379 (44.3)
Male	476 (55.7)
Type of stroke (ICD-10), *n (%)*	
I60	0 (0)
I61	33 (3.4)
I62	0 (0)
I63	674 (69.3)
I64	0 (0)
G45	148 (15.2)
Recurrence, *n (%)*	56 (6.6)
Barthel Index, *mean (SD)*	82.97 (22.72)
Barthel Index, *n (%)*	
0–24	16 (1.9)
25–49	66 (7.7)
50–74	118 (13.8)
75–99	269 (31.5)
100	336 (39.3)
Missing	50 (5.8)
Modified Rankin Scale, *median*	2
Modified Rankin Scale, *n (%)*	
0	88 (9.1)
1	195 (20.1)
2	255 (26.2)
3	225 (23.2)
4	92 (9.5)
EQ-5D-5L, *mean (SD)*	0.78 (0.27)
EQ-5D-5L VAS, *mean (SD)*	67.67 (21.15)
Stroke Impact Scale, *mean (SD)*	
Strength	69.84 (23.25)
Memory & thinking	84.28 (18.32)
Emotion	71.94 (16.89)
Communication	87.9 (16.42)
ADL/IADL	82.82 (21.38)
Mobility	81.6 (21.93)
Hand function	75.8 (29.45)
Participation & role function	72.04 (26.64)
Stroke Impact Scale VAS, *mean (SD)*	69.89 (21.03)

*I60* Subarachnoid haemorrhage, *I61* Intracerebral haemorrhage,

*I62* Other non-traumatic intracerebral haemorrhage, *I63* Ischemic stroke,

*I64* Stroke, not described as haemorrhage or infarction,

*G45* Transient ischemic attack, *VAS* Visual Analogue Scale,

*ADL/IADL* Activities of daily living/ instrumental activities of daily living

The proportion of at least one dimension missing in the 5L ranges from 3.2% (t_3_) to 3.4% (t_1_, t_2_). The completion rate of the 5L is > 95% for all time points. For the SIS, the proportion of at least one item missing per domain, has a range between 4.8% (t_3_) in the communication domain and 28.7% (t_2_) in the domain concerning participation & role function. For the overall SIS the completion rate is < 50% at all follow-ups. The domains with the highest and lowest rate of completion at all time points are communication (93.9− 95.2%) and participation & role function (71.3%− 72.3%), respectively (Table 2). The most frequently missing values on an item level are concerning strength in the most affected foot/ankle (23.4 − 24.9%), strength in the primarily affected leg (20.6 − 22%), participation in religious activities (19.6 − 21%), strength of grip in the most affected hand (15.1 − 16.6%) and strength in the most affected arm (15 − 16.1%) (Supplementary Table 1, Additional File 1). Overall, the completion rate of both VAS is similarly high. However, the EQ VAS completion rate is slightly higher than the SIS EQ VAS across all time points (t_1_: 96.96% vs. 94.98%, t_2_: 98.13% vs. 96.14%).

**Table 2:** Feasibility of the 5L and the SIS for t_1_ – t_3_

Measure/ dimension/ domain	3 months (t_1_)			6 months (t_2_)			12 months (t_3_)		
	≥ one item missing, *n* (%)	Missing, *n* (%)	Completion rate, %	≥ one item missing, *n* (%)	Missing, *n* (%)	Completion rate, %	≥ one item missing, *n* (%)	Missing, *n* (%)	Completion rate, %
*n* = 855									
5L	29 (3.4)		96.6	17 (3.4)		96.6	27 (3.2)		96.4
5L MO		12 (1.4)			13 (1.5)			11 (1.3)	
5L SC		7 (0.8)			11 (1.3)			10 (1.7)	
5L UA		9 (1.1)			12 (1.4)			11 (1.3)	
5L PD		10 (1.2)			11 (2.9)			14 (1.6)	
5L AD		7 (0.8)			8 (0.9)			6 (0.7)	
*n* = 856									
SIS			45.1			44.9			46.1
SIS strength	226 (26.4)		73.6	229 (26.7)		73.3	216 (25.2)		74.8
SIS memory & thinking	60 (7)		92.1	63 (7.4)		92.6	56 (6.5)		93.5
SIS emotion	96 (11.2)		88.8	94 (11)		89	105 (12.3)		87.7
SIS communication	48 (5.6)		94.4	52 (6.1)		93.9	41 (4.8)		95.2
SIS ADL/IADL	86 (10)		90	85 (10)		90.1	102 (11.9)		88.1
SIS mobility	58 (6.8)		93.2	78 (9.1)		90.9	67 (7.8)		92.2
SIS hand function	101 (11.8)		88.2	118 (13.8)		86.2	111 (13)		87
SIS participation & role function	237 (27.7)		72.3	246 (28.7)		71.3	239 (27.9)		72

*5L* EQ-5D-5L, *MO* Mobility, *SC* Self-Care, *UA* Usual Activities, *PD* Pain/Discomfort, *AD* Anxiety/Depression, *SIS* Stroke Impact Scale

**Fig. 1:**
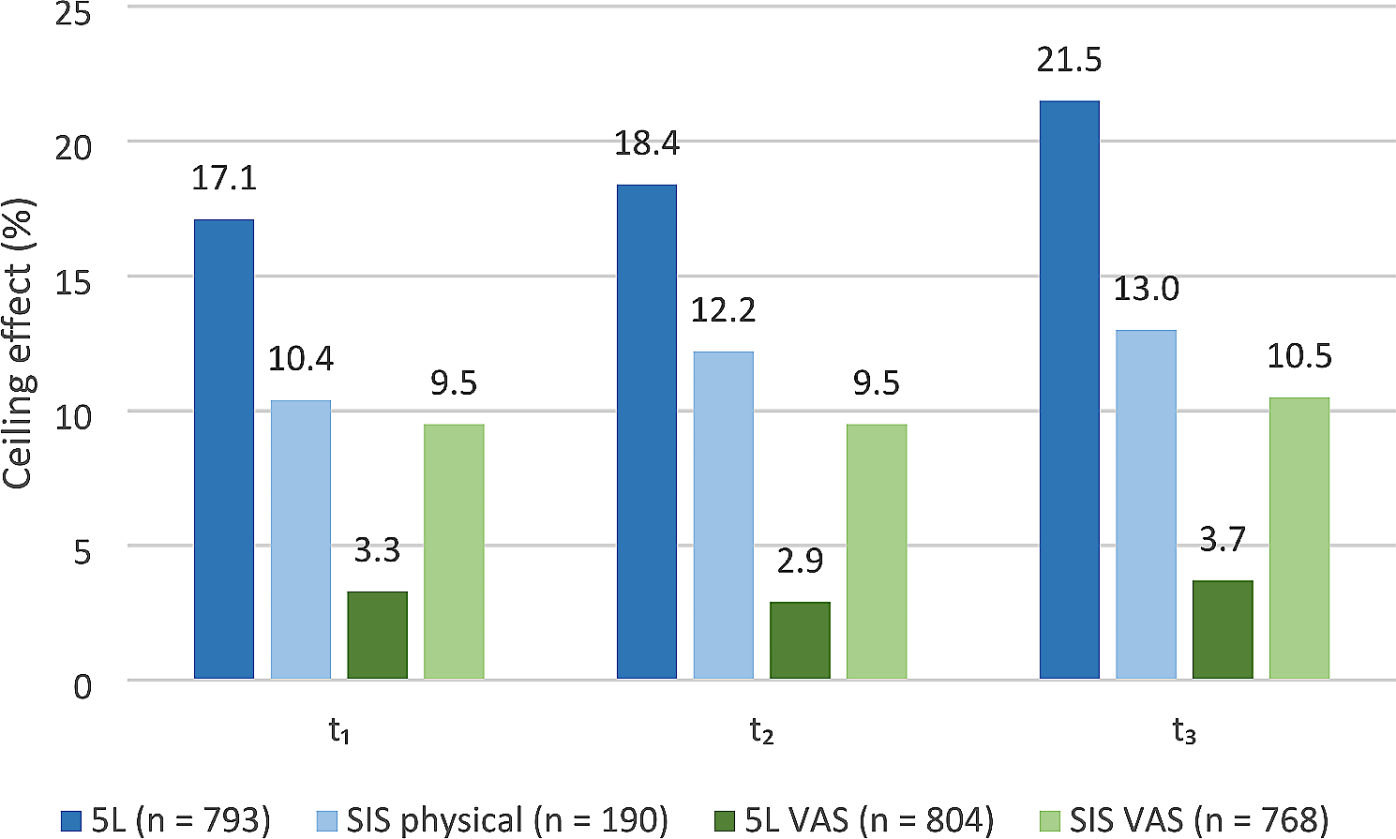
Ceiling effects of the 5L index, 5L VAS, SIS physical domain and SIS

VAS for the time point points t_1_ – t_3_. *5L* EQ-5D-5L, *SIS* Stroke Impact Scale, VAS Visual Analogue Scale.

Ceiling effects are detectable in the 5L, as the 15% threshold is exceeded at t_1_ (17.1%), t_2_ (18.4%) and t_3_ (21.5%). For each time point, the proportion of patients reporting the highest possible health status as measured by the 5L is higher in comparison to the SIS physical function domain, the SIS VAS and the EQ VAS. Although, the respective percentage of patients with a maximum score in the SIS physical function domain increases over time, the percentages at t_1_ (10.4%), t_2_ (12.2%) as well as t_3_ (13%) indicate no ceiling effects (Fig. 1). The SIS hand function domain indicates on average the highest ceiling effect with 31.3% at each time point. The only domain that does not demonstrate ceiling effects was SIS emotion (Supplementary Table 2, Additional File 1). Although ceiling effects are detected for neither VAS measure, the SIS VAS shows a higher proportion of patients reporting a score of 100 at each measured time point. For all measures and domains no floor effects are identified, as the proportion of patients reporting the worst (health) status is < 5% (Supplementary Fig. 1, Additional File 1).

**Table 3:** Responsiveness of the 5L index and the SIS physical domain

Measure	Improved Barthel Index				Deteriorated Barthel Index			
	*n*	Mean difference	ES	SRM	*n*	Mean difference	ES	SRM
3 months (t_1_) to 6 months (t_2_)								
5L index	56	0.07*	0.21	0.34	8	-0.15	-0.66	-0.64
SIS physical	12	8.84*	0.31	0.71	2	-16.3	-1.61	-2.38
3 months (t_1_) to 12 months (t_3_)								
5L index	87	0.06	0.16	0.21	16	-0.27*	-0.15	-0.97
SIS physical	17	3.35	0.12	0.32	4	-24.53	-0.57	-0.88
6 months (t_2_) to 12 months (t_3_)								
5L index	33	0.06*	0.16	0.24	12	-0.18	-0.42	-0.47
SIS physical	5	5.77	0.17	0.63	5	-20.08	-1.01	-0.88

*ES* Effect Size, *SRM* Standardized Response Mean, *5L* EQ-5D-5L, *SIS* Stroke Impact Scale,

*VAS* Visual Analogue Scale, * *p* < 0.05

Both instruments demonstrate small to moderate ES and SRM for all time point comparisons in patients that improved (Table 3). When directly comparing the 5L index and the SIS physical function domain, the latter predominantly reveals higher ES and SRM in improved patients as well as in patients that deteriorated. In patients that improved, solely the SIS physical function domain from 3 to 6 months and from 6 to 12 months demonstrates moderate effects (SRM = 0.71, SRM = 0.63, respectively). Results for ES and SRM in patients that deteriorated are of negative value. The SIS physical function domain shows large ES and SRM in patients that deteriorated. For the 5L index all changes indicate small to moderate ES, except for the deterioration from 3 to 12 months, which is large (SRM = -0.97). ES and SRM of the EQ VAS and the SIS VAS indicate small ES for patients that improved as well as patients that deteriorated in the BI. The EQ VAS predominantly proves higher ES and SRM than the SIS VAS, especially in patients that improved in terms of the BI (Supplementary Table 3, Additional File 1).

**Table 4:** Known-groups validity of the 5L and the SIS for different groups

Dimension	Sex^a^ *p*-value	Age groups^b^ H (df)	Diagnosis^b^ H (df)	mRS^b^ H (df)	BI^b, c^ H (df)
5L index (*n* = 783)	< 0.001	51.67 (9)**	17.62 (2)**	90.17 (4)**	84.8 (2)**
SIS physical (*n* = 190)	< 0.001	43.391 (9)**	16.73 (2)**	39.06 (4)**	28.04 (2)**
5L VAS (*n* = 804)	< 0.001	41.73 (9)**	18.65 (2)**	81.43 (4)**	63.45 (2)**
SIS VAS (*n* = 768)	< 0.001	23.15 (9)*	42.44 (2)**	103.91 (4)**	49.99 (2)**
5L AD (*n* = 783)	< 0.001	16.05 (9)	0.59 (2)	21.68 (4)**	20.22 (2)**
SIS emotion (*n* = 190)	< 0.05	12.75 (9)	1.92 (2)	7.15 (4	7.29 (2)*
5L UA (*n* = 783)	< 0.001	54.45(9)**	35.41 (2)**	142.28 (4)**	111.88 (2)**
SIS ADL/IADL (*n* = 190)	< 0.001	45.08 (9)**	14.43 (2)**	38.83 (4)**	32.03 (2)**
5L MO (*n* = 783)	< 0.001	70.97 (9)**	13.71 (2)*	80.77 (4)**	96.76 (2)**
SIS mobility (*n* = 190)	< 0.001	46.45 (9)**	8.42 (2)*	30.64 (4)**	26.31 (2)**

*df* degrees of freedom, *BI* Barthel Index *5L* EQ-5D-5L, *SIS* Stroke Impact Scale,

*VAS* Visual Analogue Scale, *AD* Anxiety/Depression, *UA* Usual Activities, *MO* Mobility,

* *p* < 0.05, ** *p* < 0.001

^a^ Wilcoxon rank-sum W

^b^ Kruskal-Wallis H

^c^ for the Barthel Index: 5L index, 5L AD, 5L UA, 5L MO *n* = 737; 5L VAS *n* = 758; SIS physical, SIS emotion, SIS ADL/IADL, SIS mobility *n* = 182; SIS VAS *n* = 723

The median differences of the 5L index, UA, MO and VAS between categories of the mRS, BI, type of stroke, age and sex are statistically significant (*p* < 0.05); proving their known-groups validity. Similarly, the content-related domains of the SIS (physical function, ADL/IADL, mobility) show known-groups validity (*p* < 0.05). However, known-groups validity of the 5L and SIS is partly compromised because of results in AD and in the emotion domain. The respective analyses of median differences between age groups and diagnosis do not result in statistical significance (*p* > 0.05) (Table 4). Post-hoc tests indicate differences between groups with larger differences in mRS, BI and age groups as well as differences between confirmed stroke diagnosis (I63, I61) and TIA diagnosis (G45) (data available upon request).

## Discussion

The aim of this study was to compare the measurement properties of the 5L in comparison to the SIS in a cohort of mildly to moderately affected stroke and TIA patients undergoing case management. Performance was assessed in terms of feasibility, ceiling and floor effects, responsiveness and known-groups validity. To the best of our knowledge this is the first study to compare the measurement properties of the 5L and the SIS.

The 5L index indicates excellent feasibility. Similar results have been found in previous research [20, 63]. However, it should be kept in mind that the reminding service of a study nurse might have decreased the proportion of missing values in the 5L and in the SIS. When compared to the feasibility of the EQ-5D-3L in stroke patients, the current analysis indicates improvement in terms of missing values which aligns with prior research [20, 64]. It might be explained by the extension of response levels. Evidence shows that three-point scales does not always allow participants to adequately express their feelings [65]. With regard to the EQ measures, the 5L might enable better engagement with the task to reflect the patients´ true HRQoL status. Higher rates of missingness in the SIS might be explained by the length of the SIS. Prior research has suggested that the length of the questionnaire may be a substantial patient burden; resulting in the simplification of the measure to a short form [66]. Besides that, the moderate feasibility results of the SIS support prior evidence of measurement properties [67, 68]. The work of Caël and colleagues (2015) indicates that items with the most frequently missing values are concerning religion, work and recreational activity. This is similar to presented results. Much alike this study, the sample includes TIA patients, in which symptoms usually resolve within ≤ 24 h [69]. Thus, physical consequences such as strength and hand function might not be of relevance leading to missing values. Additionally, it is no surprise that around 20% of patients did not answer the item concerning religious participation, as in 2018 around 27% of the German population declared to have no religious beliefs [70]. Although selection bias was ruled out by comparison of completers and non-completers, the above-mentioned results and prior literature suggests that missingness can nonetheless be influenced by other external factors not assessed in our study. Despite lacking comparable research, the EQ VAS and the SIS VAS show high completion rates across all time points, indicating good to excellent feasibility owing to the simplicity of the measure and its potential of quick assessment [71]. In a more detailed perspective, the EQ VAS presents with a slightly higher completion rate across all time points. Possible reasons for these marginal differences may lie within the description and content of the EQ VAS. For the EQ VAS, patients are asked to determine their health status of the corresponding day. This might present as less difficult to answer than with the SIS VAS asking patients about their current state of recovery from stroke, which spans over a longer period of time and recall of memory. Due to (temporary) cognitive impairments and difficulty remembering their post-stroke status [72], patients might be more inclined to omit answering the SIS VAS.

The evidence on ceiling and floor effects in the 5L and the SIS are inconsistent in published literature [20, 24, 25, 63, 67, 73, 74]. The current analysis, however, supports evidence of no floor effects and high ceiling effects in both measures in a cohort of elderly mild to moderately affected stroke patients. Differences in evidence might be explained by varying cohorts. The prior cited studies all excluded patients with TIA and showed lower baseline scores on the respective measures (e.g. mean 5L index: 0.526 [20]; mean SIS VAS: 51.6–63.6 [24]) as compared to this study. The current sample included patients with TIA (mean 5L index 0.78; mean SIS VAS: 69.89), for which especially physical impairments are expected to decrease or vanish altogether while psychological issues might persist [75–77]. However, most dimensions of the 5L focus on physical disability (MO, UA, SC, PD) and less on emotional factors. The results might thus imply limited suitability of the 5L for TIA patients. However, it could also be explained by a response shift often occurring in longitudinal HRQoL data as patients change their personal meaning of HRQoL or certain aspect of HRQoL over time. This is usually based on experiences or expectations that changed due to a shift in value and priorities as a result of adjustment to chronic disease [78, 79]. Diagnosis-related differences might also explain variability in ceiling effects between the SIS domains. (Long-term) symptoms of stroke are particularly individual and dependent on type and severity of stroke [80–84]. Hence, the SIS may lack sensitivity for some patients with only mild impairments and, thus, may compromise the suitability in those patients. When set against each other, the 5L indicates larger ceiling effects than the SIS physical function domain. However, ceiling effects of other SIS domains such as hand function, mobility, memory & thinking, ADL/IADL and communication exceed the results of the 5L index.

The small to moderate responsiveness of the 5L as observed in this study predominantly aligns with previous evidence [74]. However, another study has primarily found moderate responsiveness. Owing to the chosen time frame, Golicki and colleagues (2015) included patients in their acute phase [18], which is characterized by higher impairment and the most substantial progress in recovery [74]. This was also evident in the mean 5L utility at baseline (0.577). In contrast to that, the current analysis of responsiveness was based on data from three months and onwards and included TIA patients which resulted in higher 5L utility at baseline to begin with (0.78). The conclusion that the 5L index is more responsive in patients with extreme health conditions is hereby underlined [85]. To gather a comprehensive assessment of responsiveness, complete data of the first year including assessment at baseline and different time intervals than chosen in this study should be integrated in future analyses. The SIS physical function domain certainly indicates better responsiveness than the 5L index. Nonetheless, merely slight differences between the measures are observable and both measures as well as the respective VAS predominantly present with small responsiveness. Overall, the present results of the SIS indicate small to moderate responsiveness in patients that have improved according to the BI and large responsiveness in patients with deterioration in the BI. However, small sample sizes limit interpretability of the analysis. No other comparable and methodologically robust studies concerning the responsiveness of the SIS physical function domain were identified. Thus, future research should generate larger sample sizes. Besides, the current analysis merely inspects responsiveness of the physical function domain; further research including all SIS domains is needed for a comprehensive assessment of the SIS.

With regards to known-groups validity of the 5L index, EQ VAS, the 5L dimensions UA and MO discriminate between mRS, type of stroke, BI, sex and age. Besides that, with the presented results, prior research concerning the 5L index and the EQ VAS is confirmed [20]. However, prior results were merely based on descriptive data. The absence of high-quality evidence highlights the relevance of the presented results. With regard to the SIS, the domains physical function and ADL/IADL discriminated between the mRS, type of stroke, BI, sex and age. This result is contrary to prior research. In a French cohort of mildly to severely affected stroke patients, Caël and colleagues (2015) concluded that the SIS did not discriminate between type of stroke, sex and age [67]. These contradicting results might be explained by substantial ceiling effects in mildly affected stroke patients [86], making it harder for the instrument to discriminate between groups. Furthermore, with the current analysis, the hypothesis of known-groups validity of the SIS emotion domain could not be confirmed. This aligns with prior research [24].

Several limitations may influence the interpretation of the discussed findings. Generally, there is no “gold standard” for the assessment of feasibility, responsiveness and known-groups validity. Results of feasibility might be influenced by the reminding service of a study nurse, which limits the generalizability for other stroke studies. However, this service may have reduced non-response bias as a larger group of patients could have possibly been reached over all the time points. Moreover, the threshold for relevant change of the BI was based on MCID from a study by Hsieh and colleagues (2007) and adapted for a 100-point scale by the team around Golicki (2015) [18, 45]. Nonetheless, the adaptation was not further validated. Comparable studies to determine MCID for a 100-point BI are still needed. Besides that, using Cohen´s D threshold (> 0.8 large, 0.5 to 0.8 moderate, < 0.5 small) to determine responsiveness by SRM is debatable. Interpretation of responsiveness could lead to over- or underestimation [87, 88]. Furthermore, methods for assessing known-groups validity with > 2 groups (Kruskal-Wallis H test) do not allow hypothesizing a direction of comparison [89] reducing the informative power of the analysis. It is additionally mentionable that the analyzed measurement properties are not exhaustive. Other measurement properties such as construct validity (convergent and divergent validity) could not be assessed due to unavailability of required data [90–92] and should thus be considered in future research. Overall, when interpreting the results, it should be kept in mind that the patients used for this analysis are not representative of German stroke patients, as they were treated by case-management and patients with severe impairments (mRS > 5, nursing care level > 4) were excluded. A similarly designed study evaluating the HRQoL of stroke patients observed slightly worse mean SIS domain and SIS VAS scores at baseline as compared to the current study (current study SIS VAS: 69.89; similar study: 64.53) [93]. The mean 5L index and EQ VAS of the general population in Germany (0.88 and 79.45, respectively) are above the average of the observed stroke case management patients (0.78 and 67.67, respectively) [41, 94]. Furthermore, the findings on the 5L index cannot be generalized internationally as a German value set by Ludwig and colleagues (2018) was used to calculate utilities [41]. Finally, as two measures of HRQoL presenting with similar items and domains were administered, order effects cannot be ruled out [95–97]. Nevertheless, the large overall sample size is a strength of the analyses.

In conclusion, both the 5L and the SIS show adequate feasibility, responsiveness, known-group validity and ceiling effects at three to twelve months post stroke. However, the 5L appears to be slightly more suitable in terms of feasibility, ceiling effects and known-groups validity for this cohort of mildly to moderately affected stroke and TIA patients when compared to SIS domains. Nonetheless, the results of both measures indicate limited suitability for TIA patients. Further large-scale studies concerning responsiveness and known-groups validity are encouraged.

### Electronic supplementary material

Below is the link to the electronic supplementary material.

Supplementary Material 1

## Data Availability

The datasets used and analysed during the current study are available from the corresponding author on reasonable request.

## Abbreviations

5L: EQ-5D-5L
AD: EQ-5D-5L anxiety/depression
ADL: Activities of daily living
BI: Barthel Index
EQ VAS: EQ-5D-5L visual analogue scale
ES: Effect Size
HRQoL: Health-Related Quality of Life
IADL: Instrumental activities of daily living
MCID: Minimal clinically important difference
MO: EQ-5D-5L mobility
mRS: Modified Rankin Scale
PD: EQ-5D-5L pain/discomfort
SC: EQ-5D-5L self-care
SIS: Stroke Impact Scale
SRM: Standardized Response Mean
TIA: Transient ischemic attack
UA: EQ-5D-5L usual activities
