# Supplementary material for: Comparing the EQ-5D-5L and stroke impact scale 2.0 in stroke patients: an analysis of measurement properties
Source: Health Qual Life Outcomes. 2024 Jun 5;22:45. doi: 10.1186/s12955-024-02252-z (PMC11151530; doi:10.1186/s12955-024-02252-z)
Supplement: Supplementary file 1 — Supplementary Material 1 [file 12955_2024_2252_MOESM1_ESM.docx]

| **Journal** | Health and Quality of Life Outcomes |
| --- | --- |
| **Title** | Comparing the EQ-5D-5L and Stroke Impact Scale in Stroke Patients: An analysis of measurement properties |
| **Authors** | Juliana Schmidt Affiliations: School of Public Health, Bielefeld University, Bielefeld, Germany juliana.schmidt@uni-bielefeld.de 0000-0002-1828-5636  Juliane Andrea Düvel  Svenja Elkenkamp  Wolfgang Greiner |
|  |  |

**Supplementary Table 1** Missing values of SIS items for t_1_ – t_3_ (n = 856)

| SIS domain | Item | 3 months (t_1_) | |  | 6 months (t_2_) | |  | 12 months (t_3_) | |
| --- | --- | --- | --- | --- | --- | --- | --- | --- | --- |
|  |  | n | % |  | n | % |  | n | % |
| Strength | a | 131 | 15.3 |  | 138 | 16.1 |  | 128 | 15.0 |
|  | b | 135 | 15.8 |  | 142 | 16.6 |  | 133 | 15.5 |
|  | c | 188 | 22.0 |  | 187 | 21.8 |  | 176 | 20.6 |
|  | d | 213 | 24.9 |  | 213 | 24.9 |  | 200 | 23.4 |
| Memory & thinking | a | 17 | 2.0 |  | 17 | 2.0 |  | 22 | 2.6 |
|  | b | 17 | 2.0 |  | 23 | 2.7 |  | 22 | 2.6 |
|  | c | 22 | 2.6 |  | 20 | 2.3 |  | 20 | 2.3 |
|  | d | 19 | 2.2 |  | 24 | 2.8 |  | 23 | 2.7 |
|  | e | 23 | 2.7 |  | 20 | 2.3 |  | 27 | 3.2 |
|  | f | 22 | 2.6 |  | 25 | 2.9 |  | 19 | 2.2 |
|  | g | 23 | 2.7 |  | 22 | 2.6 |  | 22 | 2.6 |
|  | h | 22 | 2.6 |  | 23 | 2.7 |  | 28 | 3.3 |
| Emotion | a | 24 | 2.8 |  | 18 | 2.1 |  | 21 | 2.5 |
|  | b | 25 | 2.9 |  | 30 | 3.5 |  | 19 | 2.2 |
|  | c | 32 | 3.7 |  | 27 | 3.2 |  | 34 | 4.0 |
|  | d | 26 | 3.0 |  | 20 | 2.3 |  | 20 | 2.3 |
|  | e | 21 | 2.5 |  | 24 | 2.8 |  | 18 | 2.1 |
|  | f | 32 | 3.7 |  | 30 | 3.5 |  | 29 | 3.4 |
|  | g | 27 | 3.2 |  | 30 | 3.5 |  | 22 | 2.6 |
|  | h | 30 | 3.5 |  | 34 | 4.0 |  | 30 | 3.5 |
|  | i | 18 | 2.1 |  | 24 | 2.8 |  | 13 | 1.5 |
| Communication | a | 17 | 2.0 |  | 22 | 2.6 |  | 13 | 1.5 |
|  | b | 19 | 2.2 |  | 24 | 2.8 |  | 15 | 1.8 |
|  | c | 16 | 1.9 |  | 22 | 2.6 |  | 13 | 1.5 |
|  | d | 20 | 2.3 |  | 29 | 3.4 |  | 19 | 2.2 |
|  | e | 22 | 2.6 |  | 25 | 2.9 |  | 18 | 2.1 |
|  | f | 19 | 2.2 |  | 19 | 2.2 |  | 17 | 2.0 |
|  | g | 17 | 2.0 |  | 20 | 2.3 |  | 11 | 1.3 |
| ADL/IADL | a | 19 | 2.2 |  | 18 | 2.1 |  | 14 | 1.6 |
|  | b | 18 | 2.1 |  | 21 | 2.5 |  | 14 | 1.6 |
|  | c | 21 | 2.5 |  | 22 | 2.6 |  | 15 | 1.8 |
|  | d | 37 | 4.3 |  | 32 | 3.7 |  | 34 | 4.0 |
|  | e | 20 | 2.3 |  | 21 | 2.5 |  | 18 | 2.1 |
|  | f | 17 | 2.0 |  | 18 | 2.1 |  | 22 | 2.6 |
|  | g | 25 | 2.9 |  | 15 | 1.8 |  | 21 | 2.5 |
|  |  | 19 | 2.2 |  | 23 | 2.7 |  | 20 | 2.3 |
|  | i | 25 | 2.9 |  | 22 | 2.6 |  | 29 | 3.4 |
|  | j | 17 | 2.0 |  | 23 | 2.7 |  | 15 | 1.8 |
|  | k | 27 | 3.2 |  | 27 | 3.2 |  | 18 | 2.1 |
|  | l | 28 | 3.3 |  | 29 | 3.4 |  | 28 | 3.3 |
| Mobility | a | 18 | 2.1 |  | 20 | 2.3 |  | 20 | 2.3 |
|  | b | 23 | 2.7 |  | 19 | 2.2 |  | 20 | 2.3 |
|  | c | 23 | 2.7 |  | 24 | 2.8 |  | 20 | 2.3 |
|  | d | 21 | 2.5 |  | 32 | 3.7 |  | 26 | 3.0 |
|  | e | 18 | 2.1 |  | 23 | 2.7 |  | 26 | 3.0 |
|  | f | 21 | 2.5 |  | 25 | 2.9 |  | 20 | 2.3 |
|  | g | 20 | 2.3 |  | 23 | 2.7 |  | 23 | 2.7 |
|  | h | 22 | 2.6 |  | 21 | 2.5 |  | 23 | 2.7 |
|  | i | 20 | 2.3 |  | 22 | 2.6 |  | 20 | 2.3 |
|  | j | 17 | 2.0 |  | 19 | 2.2 |  | 18 | 2.1 |
| Hand function | a | 85 | 9.9 |  | 93 | 10.9 |  | 89 | 10.4 |
|  | b | 88 | 10.3 |  | 99 | 11.6 |  | 88 | 10.3 |
|  | c | 84 | 9.8 |  | 97 | 11.3 |  | 90 | 10.5 |
|  | d | 90 | 10.5 |  | 97 | 11.3 |  | 89 | 10.4 |
|  | e | 81 | 9.5 |  | 88 | 10.3 |  | 79 | 9.2 |
| Participation &  role function | a | 105 | 12.3 |  | 91 | 10.6 |  | 85 | 9.9 |
|  | b | 69 | 8.1 |  | 56 | 6.5 |  | 66 | 7.7 |
|  | c | 57 | 6.7 |  | 52 | 6.1 |  | 63 | 7.4 |
|  | d | 72 | 8.4 |  | 66 | 7.7 |  | 77 | 9.0 |
|  | e | 66 | 7.7 |  | 56 | 6.5 |  | 75 | 8.8 |
|  | f | 168 | 19.6 |  | 173 | 20.2 |  | 180 | 21.0 |
|  | g | 47 | 5.5 |  | 45 | 5.3 |  | 56 | 6.5 |
|  | h | 45 | 5.3 |  | 39 | 4.6 |  | 53 | 6.2 |
|  | i | 45 | 5.3 |  | 36 | 4.2 |  | 52 | 6.1 |

*SIS* Stroke Impact Scale, *ADL/IADL* (instrumental) activities of daily living

**Supplementary Table 2** Ceiling and floor effects of the SIS domains for t_1_ – t_3_ (n = 190)

| SIS domain | 3 months (t_1_) | |  | 6 months (t_2_) | |  | 12 months (t_3_) | |
| --- | --- | --- | --- | --- | --- | --- | --- | --- |
|  | Ceiling  % | Floor  % |  | Ceiling  % | Floor  % |  | Ceiling  % | Floor  % |
| Memory & thinking | 23.9 | 0.0 |  | 26.5 | 0.0 |  | 26.1 | 0.0 |
| Emotion | 3.5 | 0.0 |  | 3.9 | 0.0 |  | 4.8 | 0.0 |
| Communication | 32.2 | 0.0 |  | 30.4 | 0.0 |  | 27.8 | 0.0 |
| ADL/IADL | 22.2 | 0.0 |  | 22.6 | 0.0 |  | 23.0 | 0.0 |
| Mobility | 24.8 | 0.0 |  | 26.5 | 0.0 |  | 26.1 | 0.0 |
| Hand function | 31.3 | 2.6 |  | 31.3 | 4.3 |  | 31.3 | 2.6 |
| Participation &  role function | 17.0 | 0.0 |  | 23.0 | 0.4 |  | 21.3 | 0.0 |
| Physical | 10.4 | 0.0 |  | 12.2 | 0.0 |  | 13.0 | 0.0 |

*SIS* Stroke Impact Scale, *ADL/IADL* (instrumental) activities of daily living

**Supplementary figure 1** Floor effects of the 5L index, 5L VAS, SIS physical domain and SIS
VAS for the time point points t_1_ – t_3_
*5L* EQ-5D-5L, *SIS* Stroke Impact Scale, *VAS* Visual Analogue Scale

**Supplementary Table 3** Responsiveness of the 5L VAS and the SIS VAS

| Measure | Improved Barthel Index | | | | |  | Deteriorated Barthel Index | | | | | |  |
| --- | --- | --- | --- | --- | --- | --- | --- | --- | --- | --- | --- | --- | --- |
|  | n | Mean difference | | ES | SRM | | |  | n | Mean difference | ES | SRM | |
| 3 months (t_1_) to 6 months (t_2_) | | | | | | | | | | | | | |
| 5L  VAS | 63 | | 7* | 0.32 | 0.39 | | |  | 9 | -8 | -0.3 | -0.43 | |
| SIS  VAS | 52 | | 5* | 0.19 | 0.34 | | |  | 9 | -11 | -0.36 | -0.41 | |
| 3 months (t_1_) to 12 months (t_3_) | | | | | | | | | | | | | |
| 5L  VAS | 94 | | 8.2** | 0.36 | 0.45 | | |  | 15 | -4.4 | -0.16 | -0.2 | |
| SIS  VAS | 84 | | 6* | 0.25 | 0.32 | | |  | 16 | -8.3 | -0.29 | -0.3 | |
| 6 months (t_2_) to 12 months (t_3_) | | | | | | | | | | | | | |
| 5L  VAS | 33 | | 6.7* | 0.28 | 0.49 | | |  | 11 | -10 | -0.46 | -0.49 | |
| SIS  VAS | 34 | | 6* | 0.26 | 0.42 | | |  | 12 | -8 | -0.48 | -0.5 | |

*ES* Effect Size, *SRM* Standardized Response Mean, *5L* EQ-5D-5L, *SIS* Stroke Impact Scale,
*VAS* Visual Analogue Scale, * p < 0.05, ** p < 0.001
